# A Sports Nutrition Perspective on the Impacts of Hypoxic High-Intensity Interval Training (HIIT) on Appetite Regulatory Mechanisms: A Narrative Review of the Current Evidence

**DOI:** 10.3390/ijerph19031736

**Published:** 2022-02-02

**Authors:** Chung-Yu Chen, Chun-Chung Chou, Ke-Xun Lin, Toby Mündel, Mu-Tsung Chen, Yi-Hung Liao, Shiow-Chwen Tsai

**Affiliations:** 1 Department of Exercise and Health Sciences, University of Taipei, Taipei City 111, Taiwan; fish0510@gmail.com; 2 Physical Education Office, National Taipei University of Technology, Taipei City 106, Taiwan; longer0206@gmail.com; 3 Department of Exercise and Health Science, National Taipei University of Nursing and Health Sciences, Taipei City 112, Taiwan; andy121317062@gmail.com; 4 School of Sport, Exercise and Nutrition, Massey University, Palmerston North 4442, New Zealand; T.Mundel@massey.ac.nz; 5 Department of Food and Beverage Management, Shih Chien University, Taipei City 104, Taiwan; bruce@g2.usc.edu.tw; 6 Institute of Sports Sciences, University of Taipei, Taipei City 111, Taiwan

**Keywords:** ghrelin, leptin, PYY, GLP-1, high altitude

## Abstract

High-intensity interval training (HIIT) and low-oxygen exposure may inhibit the secretion of appetite-stimulating hormones, suppress appetite, and inhibit dietary intake. Physiological changes affecting appetite are frequent and include appetite hormone (ghrelin, leptin, PYY, and GLP-1) effects and the subjective loss of appetite, resulting in nutritional deficiencies. This paper is a narrative review of the literature to verify the HIIT effect on appetite regulation mechanisms and discusses the possible relationship between appetite effects and the need for high-intensity exercise training in a hypoxic environment. We searched MEDLINE/PubMed and the Web of Science databases, as well as English articles (gray literature by Google Scholar for English articles) through Google Scholar, and the searched studies primarily focused on the acute effects of exercise and hypoxic environmental factors on appetite, related hormones, and energy intake. In a general normoxic environment, regular exercise habits may have accustomed the athlete to intense training and, therefore, no changes occurred in their subjective appetite, but there is a significant effect on the appetite hormones. The higher the exercise intensity and the longer the duration, the more likely exercise is to cause exercise-induced appetite loss and changes in appetite hormones. It has not been clear whether performing HIIT in a hypoxic environment may interfere with the exerciser’s diet or the nutritional supplement intake as it suppresses appetite, which, in turn, affects and interferes with the recovery efficiency after exercise. Although appetite-regulatory hormones, the subjective appetite, and energy intake may be affected by exercise, such as hypoxia or hypoxic exercise, we believe that energy intake should be the main observable indicator in future studies on environmental and exercise interventions.

## 1. Introduction

Exercise training is an intervention used by athletes to enhance their performance [1,2,3]. In addition, coaches also frequently plan different types of energy system development training (ESD training) according to the athletes’ competition demands, including special training modes for enhancing specific energy systems, such as the ATP–PCr system, the anaerobic glycolytic system, and the aerobic glycolytic system [4,5,6,7]. Currently, high-intensity interval training (HIIT) or high-intensity interval exercise (HIIE) is considered an effective and time-saving exercise strategy to improve endurance sports performance [8]. HIIT is widely used in athletic populations to enhance the efficiency of various energy systems and to promote sports performance [9,10]. On the other hand, sports scientists also use a hypoxic environment, in combination with specific exercise training modes, to promote sports performance. HIIT is increasingly becoming a common practice in the athletic training community. Performing exercise in a hypoxic environment has the benefits of increasing VO_2_max, reducing post-exercise blood lactate, decreasing the exercise exhaustion time [11], increasing skeletal muscle capillary density, and improving vasodilatation [12]. Therefore, the combination of HIIT in a hypoxic environment is very effective and helpful in enhancing endurance exercise performance for athletes.

However, there are very limited studies on the interval training effects on exercise training, and the benefits. Studies have found that exercise at greater than 60% VO_2_max may induce a decrease in appetite and suppress the secretion of appetite-promoting hormones [13]. Similarly, low ambient oxygen exposure (FiO_2_ ≈ 11.5–13.8%) has also been found to suppress appetite and dietary intake [14,15,16]. Current research evidence also suggests that athletes should take appropriate post-exercise nutritional supplementation during the post-exercise recovery period to promote sports performance, stimulate muscle glycogen replenishment, and attenuate exercise-induced muscle damage [17,18,19,20]. After exercise, the muscle state remains, in many ways, similar to the metabolic stresses and challenges experienced during exercise; however, if an appropriate recovery strategy is not performed, such a stress state can further deteriorate and may affect subsequent exercise recoveries and adaptation benefits. Intramuscular high-energy phosphate levels are depleted, glycogen levels are reduced, and exercise-induced cortisol remains at relatively high levels after exercise, which means there is substantial catabolic activity [21,22]. Moreover, other catabolic hormones (i.e., epinephrine and norepinephrine) remain elevated for 30–60 min and gradually return to pre-exercise levels [23]. Furthermore, free radicals produced during exercise are present and result in muscle cell integrity, causing damage for many hours after exercise [24,25]. Therefore, it would be critical for ingesting, with the appropriate nutritional supplementation, better postexercise adaptations. Although the post-exercise dietary supplementation is important to promote recovery, the specific training conditions that combine hypoxia and exercise may affect the willingness of athletes to consume sufficient dietary and nutritional supplements. This may affect and interfere with post-exercise recovery efficiency. On the other hand, according to existing evidence, high-intensity and interval training has been reported to cause negative effects on appetite and also negatively regulate hormonal responses controlling appetite [26,27], but most of the related investigations were conducted in a regular laboratory setting (i.e., sea-level conditions). However, there are only a few studies focusing on the acute impacts of HIIT on appetite regulation under hypoxic conditions.

Therefore, this narrative review is a compilation of the existing literature on the effects of the acute exercise challenge, hypoxic exposure, the combined hypoxic environment, and the acute interval exercise challenge on the subjective appetite and the physiological mechanisms of appetite regulation. A complete compilation of the current knowledge in this field, through a review of the relevant literature, is presented. This review has three main objectives: (1) the effects of high-intensity exercise challenges on appetite regulation; (2) the effects of hypoxic exposure and high-altitude environments on appetite regulation; and (3) the effects of acute hypoxia, combined with interval exercise challenges, on appetite, the related regulatory hormones, and energy intake. Currently, some studies have demonstrated that high-intensity exercise and hypoxia exposure may suppress appetite hormone secretion, suppress appetite, and suppress dietary intake [13,14,28,29,30,31,32,33,34]. However, when athletes need to perform high-intensity exercise training in a hypoxic environment, how one provides effective sports nutrition supplementation is an important sports nutrition issue to be explored. Therefore, we attempt to integrate the current published evidence regarding the potential relationships and appetite-regulatory physiological mechanisms when performing HIIT in a hypoxic environment.

## 2. Study Type and Search Strategy

**Figure 1:**
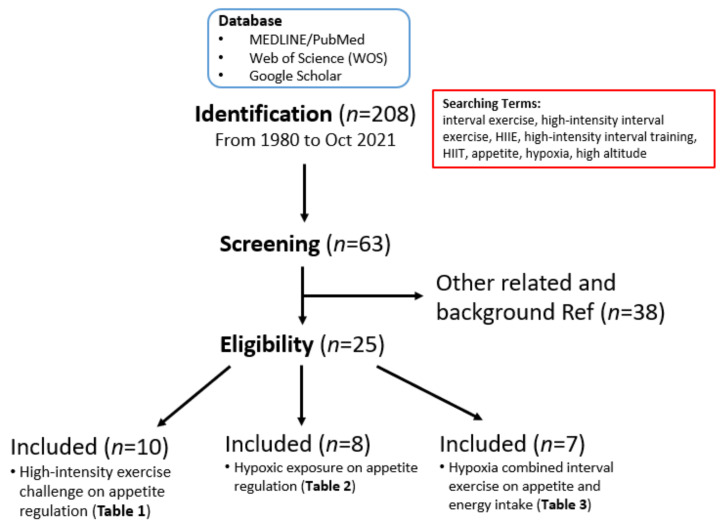
Search strategy and research article selection process. This flowchart describes the article search for this narrative literature review and the process of selecting articles on HIIT, hypoxia, appetite regulation, other research utilization, evidence-based practice, and knowledge translation for inclusion in the scoping review.

We conducted a narrative literature review to examine the potential acute changes in the effects of exercise, as well as hypoxic environmental factors, on appetite and the appetite-regulatory hormones. We searched the scientific literature in MEDLINE/PubMed and the Web of Science (WOS) databases, as well as through Google Scholar for articles published in English from 1980 through to October 2021. Multiple combinations of MeSH terms, entry terms, and keywords (i.e., interval exercise, high-intensity interval exercise, HIIE, high-intensity interval training, HIIT, appetite, hypoxia, and high altitude) were used. All descriptors were searched using the Boolean operators “OR” and “AND” to obtain a comprehensive search. Two hundred and eight articles of potential interest were found. After reading the abstracts, 63 articles were selected for the preparation of this narrative review, and 25 of them were included for the detailed review and for constructing the summary tables (see Figure 1 for the selection flow chart).

Considering that appetite hormones are decisive factors affecting nutritional intake, we selected articles related to these topics and appetite-related hormonal changes. Furthermore, we selected articles that analyzed the effects of high-intensity exercise in a hypoxic environment on appetite. Although we cannot exclude that there may be other factors (e.g., medical conditions, mental health, dietary patterns, etc.) influencing appetite regulation, the present review focuses on the literature related to the hormonal and subjective appetite regulation responses to exercise and hypoxic stimulation. We used the current findings obtained from both human and animal studies to illustrate the proposed physiological mechanisms and the clinical effects of exercise and hypoxic environmental factors on appetite, the related hormones, and energy intake.

## 3. The Impact of the High-Intensity Exercise Challenge on Appetite Regulation

Hormones play an important role in the regulation of the body’s internal balance. For example, endurance exercise has been shown to improve body composition, blood pressure, and cardiovascular function more than resistance training [35,36,37]. In addition, exercise also alters hormonal regulation; for example, the concentrations of adrenaline, insulin, cortisol, and growth hormones are increased during exercise in response to physical challenges [38]. In addition, HIIT has also been shown to increase circulating appetite inhibitory hormones (i.e., PYY, GLP-1, etc.) and, thereby, lead to a subjective loss of appetite. This physiological phenomenon is known as exercise-induced anorexia symptoms [26,27]. The relevant literature on the effects of high-intensity exercise challenge on appetite regu-lation is presented in Table 1.

Deighton and Stensel reported that exercise with ≥60% of VO_2_max strongly suppressed appetite during and shortly after exercise, whether it was running, cycling, swimming, or resistance exercise [13]. In addition, some studies have begun to investigate HIIT, which has become popular in recent years, and found that it also appears to have a suppressive effect on subjective appetite [28]. A comprehensive review of recent studies found that participants with regular exercise habits did not show changes in their subjective appetite. Hormonal effects are seen in appetite. Holliday and Blannin studied 12 participants, aged 21 ± 2 years, with a body mass index (BMI) of 21 ± 1.6 kg/m^2^, and performed the following four different trials: (1) rest (sedentary control trial); (2) 80% VO_2_max for 15 min cycling exercise; (3) 80% VO_2_max for 30 min cycling exercise; and (4) 80% VO_2_max for 45 min cycling exercise. The results showed no difference in subjective appetite and peptide YY (PYY) among trials. The ghrelin concentration was observed as significantly decreased immediately post-exercise compared to REST and remained below REST at 20 min post-exercise. The glucagon-like peptide-1 (GLP-1) concentration decreased significantly compared to REST. GLP-1 concentrations increased by 36%, 49%, 64%, and 54% immediately after 15 min of continuous exercise, with a 900% reduction in relative energy intake compared to REST [29]. Hazell and colleagues, recruiting 10 males with regular exercise regimes (VO_2_max: 46.8 ± 4.8 mL/kg/min), completed four different trials: (1) moderate-intensity continuous training (MICT: 65% VO_2_max for 30 min); (2) high-intensity continuous training (HICT: 85% VO_2_max for 30 min); (3) sprint interval training (SIT: 6*30 s with 4 min rest); (4) sedentary (control, sedentary trial). The results showed that there was no change in their subjective appetite and GLP-1 among the four trials. However, immediately after exercise, the circulating PYY concentrations were 200% higher in HICT than in the control, and the PYY level of the SIT trials was nearly 400% higher than in the control trial [30]. In the same year, Hazell’s team published another study to compare the gender differences and reported that the subjective appetite was significantly lower by approximately ~87% in SIT and ~83% in MICT, compared to the control in females [27]. In both males and females, the overall circulating PYY and GLP-1 responses were greater in MICT and SIT, compared to the control, and the subjective appetite was lower immediately after MICT and SIT [27]. Moreover, males exhibited greater total PYY concentrations immediately after exercise compared to women. Women had a higher GLP-1 response in MICT and SIT than in the control trial. These results suggest that the total circulating PYY and GLP-1 respond differently to exercise in males and females during the 90 min after exercise, with various intensities [27].

The exercise intensity and the appetite hormonal responses appear to be positively correlated, and subjective appetite may vary by gender and training patterns, especially for participants with regular training who are not acclimated to the exercise intensity and do not perceive any differences when completing visual analog scales (VAS) of appetite. Balaguera-Cortes’ team recruited 10 healthy active men to complete three different trials, including (1) RES (resistance exercise), (2) AER (aerobic exercise), and (3) resting (CON, resting control), and reported that RES showed a 20% decrease in the acylated appetite hormone compared to CON, while PYY and leptin did not change significantly. Moreover, there was no significant difference in energy intake among AER, RES, and CON [39]. Another recent study by Charlot and Chapelot recruited 15 healthy men who exercised regularly to perform HIIE consisting of 13 sets of 90% VO_2_max exercise for 30 s and 35% VO_2_max exercise for 60 s (total exercise time: 20 min), moderate-intensity continuous exercises (MIE; 42% VO_2_ max for 40 min), and the sedentary rest control trial (REST). The results showed that no change occurred in subjective appetite and energy intake, but HIIE delayed their meal by nearly 20 min compared to MIE [40]. The results from the above studies suggest that exercise intensity has a significant effect on appetite hormonal responses and subjective appetite. Taken together, there are several different HIIT exercise modes in the current publications (e.g., high-intensity aerobic interval or sprint interval exercise), and these HIIT exercise modes have been shown to similarly elevate circulating PYY and GLP-1, suggesting acute HIIT might negatively perturb appetite.

**Table 1:** Summary of the related studies on the impacts of high-intensity exercise challenge on appetite regulation.

Authors (Years)	Subject (Human)	Experimental Design	Experimental Intervention	Dietary Control Methods	Measurement	Major Findings (Δ% Change)
Hazell et al., 2017 [30]	♦ 10 healthy males (at least 3 times strenuous exercise/ week) ♦ Age: 29 ± 6 years ♦ BMI: 23.7 ± 2.2 kg/m^2^ ♦ VO_2max_: 46.8 ± 4.8 mL/kg/min	♦ Randomized and crossover ♦ Acute	♦ MICT (65% VO_2max_) 30 min cycling exercise ♦ HICT (85% VO_2max_) 30 min cycling exercise ♦ SIT (6 * 30 s all out, 4 min active recovery) 24 min cycling exercise ♦ CTRL	N/A	♦ Appetite ♦ PYY ♦ GLP-1	♦ **Perceived appetite** ⇔ ♦ **GLP-1 (compared to baseline)** ⇔ ♦ **PYY (compared to baseline)** **Immediately post-exercise** - SIT ↑ 400% than CTRL - SIT ↑ 230% than MICT - HICT ↑ 200% than CTRL
Holliday & Blannin., 2017 [29]	♦ 12 endurance-trained males ♦ Age: 21 ± 2 years ♦ BMI: 21.0 ± 1.6 kg/m^2^ ♦ VO_2max_: 61.6 ± 6.0 mL/kg/min	♦ Counter-balanced order ♦ Acute	♦ REST ♦ 80% VO_2max,_ 15 min cycling exercise (15 MIN) ♦ 80% VO_2max,_ 30 min cycling exercise (30 MIN) ♦ 80% VO_2max,_ 45 min cycling exercise (45 MIN)	N/A	♦ Appetite ♦ Ghrelin ♦ PYY ♦ GLP-1 ♦ Subjects consumed an ad libitum meal following 60 min post exercise	♦ **Perceived appetite** ⇔ ♦ **Ghrelin** **Immediately post-exercise** - 45 MIN exercise ↓ 86% than REST - 30 MIN exercise ↓ 50% than REST - 15 MIN exercise ↓ 35% than REST - 45 MIN exercise ↓ 24% than 30 MIN exercise **20 min post-exercise** - 45 MIN exercise ↓ 52% than REST ♦ **PYY** ⇔ ♦ **GLP-1** **Immediately post-exercise** - 30 MIN exercise ↑ 18% than 15 MIN exercise - 45 MIN exercise ↑ 36% than 15 MIN exercise **20 min post-exercise** - 45 MIN exercise ↑ 49% than 15 MIN exercise **40 min post-exercise** - 45 MIN exercise ↑ 64% than 15 MIN exercise **60 min post-exercise** - 45 MIN exercise ↑ 55% than 15 MIN exercise ♦ **Post 60 min exercise, subjects consumed an ad libitum meal** **Absolute food intake** ⇔ **Relative energy intake** - 30 MIN exercise ↓ 225% than REST - 45 MIN exercise ↓ 900% than REST - 45 MIN exercise ↓ 400% than 15 MIN exercise
Hazell et al., 2017 [27]	♦ 10 healthy males Age: 28.6 ± 5.9 years BMI: 23.7 ± 2.2 kg/m^2^ VO_2max_: 46.8 ± 4.8 mL/kg/min ♦ 11 healthy females Age: 30.5 ± 7.9 years BMI: 23.5 ± 2.8 kg/m^2^ VO_2max_: 40.7 ± 5.4 mL/kg/min	♦ Randomized and crossover ♦ Acute	♦ MICT (65% VO_2max_) 30 min cycling exercise ♦ SIT (6 * 30 s all out, 4 min active recovery) 24 min cycling exercise ♦ CTRL	♦ Consumed standardized breakfast before exercise: 16.8 kJ/kg body mass	♦ Appetite ♦ PYY ♦ GLP-1	♦ **Perceived appetite** - Female MICT ↓ 83% than CTRL - Female SIT ↓ 87% than CTRL ♦ **PYY** **AUC in female and male** ⇔ **Immediately post-exercise** - Male MICT ↑ 25% than female MICT - Male SIT ↑ 27% than female MICT ♦ **GLP-1** **AUC in female and male** ⇔ **Immediately post-exercise** - Male, CTRL vs MICT vs SIT⇔ - Female MICT ↑ 53% than female CTRL - Female SIT ↑ 47% than female CTRL
Charlot et al., 2019 [40]	♦ 15 healthy males ♦ Age: 20.1 ± 2.2 years ♦ BMI: 23.5 ± 3.1 kg/m^2^ ♦ VO_2max_: 47 ± 12.2 mL/kg/min	♦ Randomized and crossover ♦ Acute	♦ HII ex (90% VO_2max_ 30s, 35% VO_2max_ 60s; 13 set) 20 min cycling exercise ♦ MIC ex (42% VO_2max_) 40 min cycling exercise ♦ REST	♦ Consumed standardized breakfast before exercise: 1908 ± 315 kJ (66% carbohydrate, 24% protein, 10% fat)	♦ Appetite ♦ Energy intake ♦ Meal request after exercise	♦ **Apetite** ⇔ ♦ **Energy intake** ⇔ ♦ **Meal request after exercise** - HII ex ↑ 19% than REST
Douglas et al., 2017 [41]	♦ 47 participants ♦ **Healthy lean** (11 males; 11 females) Age: 37.5 ± 15.2 years BMI: 22.4 ± 1.5kg/m^2^ ♦ **Healthy overweight/obese** (14 males; 11 females) Age: 45 ± 12.4 years BMI: 29.2 ± 2.9 kg/m^2^	♦ Randomized and crossover ♦ Acute	♦ Control ♦ 60 min (59 ± 4% peak oxygen uptake) treadmill exercise	♦ Dinner the day before experiment: Male: 3138 kJ Female: 2820 kJ (71% carbohydrate, 11% protein, 18% fat)	♦ Appetite ♦ Ghrelin ♦ PYY ♦ GLP-1 ♦ Energy intake	♦ **Appetite** **1.5 h post-exercise** - compared to control, ↓ - 95% CI -3.1 to -0.5 mm, *p* = 0.01 ♦ **Ghrelin** ⇔ ♦ **PYY** **During exercise** - compared to control, ↑ - 95% CI 10 to 17 pg ml−1 *p* < 0.001 **Immediately post-exercise** - Lean group ↑ 230% than overweight/obese group ♦ **GLP-1** **During exercise** - compared to control, ↑ - 95% CI 7 to 10 pmol l−1 *p* < 0.001 **Immediately post-exercise** - Overweight/obese group ↑ 73% than lean group ♦ **Energy intake** ⇔
Christ et al., 2006 [42]	♦ 11 endurance-trained male athletes ♦ Age: 31.4 ± 1.7 years ♦ BMI: 22.6 ± 0.5 kg/m^2^ ♦ VO_2peak_: 63.3 ± 2.2 mL/kg/min	♦ Randomized and crossover ♦ Acute	♦ 180 min aerobic exercise test (50% Wmax) on a cycle ergometer	♦ **LF** - 0.5 g/kg fat, 7 g/kg carbohydrate, 1.2 g/kg protein; period: 2.5 days ♦ **HF** - 0.5 g/kg fat, period: 1 day - 3.5 g/kg fat, period: 1.5 day - 7 g/kg carbohydrate, 1.2 g/kg protein; period: 2.5 days ♦ **Pre-exercise** Consumed standardized breakfast (50 g carbohydrate)	♦ Ghrelin ♦ Leptin	♦ **Ghrelin AUC** - LF ↑ 23% than HF ♦ **Leptin** **Pre-exercise** - HF ↑ 20% than LF **AUC** - HF ↑ 38% than LF
Holliday & Blannin., 2017 [43]	♦ 8 overweight participants (4 males and 4 females) ♦ Age: 34 ± 12 years ♦ BMI: 27.7 ± 1.7 kg/m^2^	♦ Randomized and crossover ♦ Acute	♦ EX (4 × 30 s adapted Wingate test) ♦ REST	♦ Consumed standardized breakfast before exercise: 415 kcal (71% carbohydrate, 10% protein, 19% fat)	♦ Appetite ♦ Ghrelin ♦ GLP-1 ♦ Energy intake	♦ **Appetite** **Immediately post-exercise** - EX ↓ 49% than REST **30 min post-exercise** - EX ↓ 53% than REST **AUC** - EX ↓ 20% than REST ♦ **Ghrelin** **Immediately post-exercise** - EX ↓ 40% than REST **30 min post-exercise** - EX ↓ 63% than REST **60 min post-exercise** - EX ↓ 52% than REST **90 min post-exercise** - EX ↓ 35% than REST **120 min post-exercise** - EX ↓ 31% than REST **AUC** - EX ↓ 42% than REST ♦ **GLP-1** AUC - EX ↑ 22% than REST ♦ **Energy intake** ⇔
Sim et al., 2014 [44]	♦ 17 overweight participants ♦ Age: 30 ± 8 years ♦ BMI: 27.7 ± 1.6 kg/m^2^ ♦ VO_2peak_: 39.2 ± 4.8 mL/kg/min	♦ Randomized and counter-balanced ♦ Acute	♦ MC (60% VO_2peak_, 30 min) ♦ HI (100% VO_2peak_ 60 s, 50% VO_2peak_ 240 s, 6 set; duration: 30 min) ♦ VHI (170% VO_2peak_ 15 s, 32% VO_2peak_ 60 s, 24 set; duration: 30 min) ♦ CON	♦ Participants consumed a standard caloric meal (Post-exercise): - First meal: 1120 kJ (61% carbohydrate, 15% protein, 30% fat) - Second meal (first meal 70 min later): ad libitum meal	♦ Appetite ♦ Ghrelin ♦ Leptin ♦ PYY ♦ Energy intake	♦ **Appetite** ⇔ ♦ **Ghrelin** **Immediately post-exercise** - VHI ↓ 39% than CON - VHI ↓ 17% than HI - VHI ↓ 27% than MC ♦ **Leptin** ⇔ ♦ **PYY** ♦ **Energy intake** - HI ↓ 19% than CON - VHI ↓ 22% than CON - VHI ↓ 16% than MC
Poon et al., 2018 [45]	♦ 11 physically inactive participants ♦ Age: 45.7 ± 7.4 years ♦ BMI: 23.5 ± 2.1 kg/m^2^ ♦ VO_2max_: 38.5 ± 5.4 mL/kg/min	♦ Randomized and crossover ♦ Acute	♦ HIIT (100% VO_2max_ 1 min, 50% VO_2max_ 1 min, 10 set; duration: 20 min) ♦ MICT (65% VO_2max_ 40 min) ♦ VICT (80% VO_2max_ 20 min)	N/A	♦ Appetite ♦ Energy intake	♦ **Appetite** ⇔ ♦ **Energy intake** ⇔
Matos et al., 2018 [46]	♦ 12 obese participants ♦ Age: 28.4 ± 2.6 years ♦ BMI: 35.5 ± 4.5 kg/m^2^	♦ Randomized and crossover ♦ Acute	♦ MICE (70% HR_max_, 20 min) ♦ HIIE (90% HR_max_ 60 s + 60 s rest, 10 set, duration: 20 min) ♦ CON	♦ 1 h before exercise: consumed 4.5 kacl * body weight (kg) (87.5% carbohydrate, 11.2% protein, 1.3% fat)	♦ Appetite ♦ GLP-1 ♦ Energy intake	♦ **Appetite** **Hunger, immediately post-exercise** - HIIE ↓ 100% than CON ♦ **GLP-1** **1 h post-exercise** - MICE ↑ 8% than CON - HIIE ↑ 4% than CON (*p* = 0.069) ♦ **Energy intake** ⇔

↑ increase; ↓ decrease; ⇔ no difference. MICT: moderate-intensity continuous training; HICT: high-intensity continuous training; SIT: sprint interval training; CTRL: control; HII ex: high-intensity interval exercises; MIC ex: moderate-intensity continuous exercises; HF: high fat; LF: low fat; EX: exercise; MC: continuous moderate-intensity exercise; HIIT: high-intensity interval training; MICE: moderate-intensity continuous exercise; VICT: vigorous-intensity continuous training; CON: control; HIIE: high-intensity interval exercise; VHI: very-high-intensity intermittent exercise.

## 4. Low Oxygen Exposure Effects on Appetite Regulation

The relevant literature on the effects of hypoxic challenge on appetite regulation is presented in Table 2. A high altitude is defined as altitudes between 1500 and 3500 m (FiO_2_ = 17.6–13.8%), very high altitudes are defined as between 3500 and 5500 m (FiO_2_ = 13.8–10.9%), and extreme altitudes are greater than 5500 m (FiO_2_ = 10.9%) [47]. In terms of the physiological responses to high altitude environments, atmospheric pressure decreases with increasing vertical altitude from low to high altitude, but the percentage of oxygen remains at 20.9%. The inhaled oxygen pressure (PiO_2_) decreases in parallel with the atmospheric pressure. This leads to a decrease in alveolar pressure, intra-arterial oxygen pressure (PaO_2_), and arterial oxygen saturation (SpO_2_), resulting in a decrease in oxygen delivery to the tissues. When the oxygen concentration in the blood is too low, peripheral chemoreceptors are stimulated in the carotid artery and aortic arch, which are sensitive to the reflex response to hypoxia [48]. Exposure to a high-altitude environment leads to decreased PaO_2_, increased adrenaline, increased heart rate, increased cardiac output, increased lactate, an altered muscle blood flow, basal metabolic rate, and even decreased VO_2_max [49].

Hypoxemia may not only affect athletic performance, but it may also lead to altitude sickness, resulting in a loss of appetite, a phenomenon known as high-altitude anorexia. To investigate the high-altitude effect on appetite regulatory hormones, Shukla and colleagues transported 30 participants who had never been to high altitudes before (adaptation group), by helicopter, to an altitude of 3600 m (FiO_2_ = 13.8%) for 48 h (HA1). The athletes were then transported, by land, to an altitude of 4300 m (FiO_2_ = 12.5%). After moving to a high altitude for 48 h (HA2a) and 7 days (HA2b), the researchers assessed changes in hunger and blood leptin concentrations for the participants [31]. The results showed that leptin increased by 54.9% and 51% in HA2a and HA2b, compared to normal levels, while hunger decreased by 34.6% and 38.4% in HA1 and HA2a, compared to normal levels. Furthermore, Shukla et al. found that leptin concentrations were about 35% higher in HA2a (7.6 + 0.6 ng/mL in the acclimatized group and 5.6 + 0.5 ng/mL in the control group, *p* < 0.01, n = 50) and the hunger feeling score was about 30% lower in HA2a compared to the same-age group (the control group) who had lived in the high mountains for more than 6 months [31]. In another study, participants were exposed to a simulated altitude of 4100 m (FiO_2_ = 13%) for 17 h under normoxic conditions using a hypoxic tent. The results showed a 52% increase in fasting leptin concentrations under hypoxic conditions, but no significant change in postprandial GLP-1 concentrations [50]. These results suggest that when humans are exposed to extreme altitudes, appetite-regulatory hormonal responses can be significantly affected, which, in turn, can have a significant impact on appetite. Not all studies show consistent findings on the leptin elevation due to hypoxic conditions. For example, some studies have reported that exposure of healthy men to hypoxia (FiO_2_ = 15.0%) for 7 h to 10 days did not significantly alter their subjective appetite or blood leptin concentrations [51,52]. Some studies have reported that hypoxic exposure may lead to a decrease in leptin concentrations [32]. For example, Morishima and Goto subjected eight healthy males (age: 21.0 ± 0.6 years; BMI: 23.4 ± 1.1 kg/m^2^) to 7 h of rest in a hypoxic chamber in (1) a normoxic rest test (FiO_2_ = 20.9%) and (2) a hypoxic rest test (FiO_2_ = 15.0%), with the first meal given at 1 h and at 4 h after entering the laboratory. The first meal given was 3117 kJ (68.4% carbohydrate, 10.1% protein, and 21.5% fat) and the second meal given was 3059 kJ (66.9% carbohydrate, 10.1% protein, and 23.0% fat) for 1 and 4 h, respectively. No significant differences in hormone GLP-1 or leptin blood levels, which are responsible for appetite regulation, were found over a 7-hour period, nor did they specifically alter subjective appetite [52].

**Table 2:** Summary of the related studies on low ambient oxygen (hypoxic) exposure effect on appetite regulation.

Authors (Years)	Subject (Human)	Experimental Design	Experimental Intervention	Dietary Control Methods	Measurement	Major Findings (Δ% Change)
Mekjavic et al., 2016 [51]	♦ 11 healthy males ♦ Age: 23.7 ± 4 years ♦ BMI: 22.4 ± 2.4 kg/m^2^ ♦ VO_2 peak_: 60.6 ± 9.5 mL/kg/min	♦ Randomized and crossover ♦ Intervention duration: 10 days	♦ Hypoxia PiO_2_ = 105.6 mmHg (2800m), 2 days PiO_2_ = 102.9 mmHg (3000m), 2 days PiO_2_ = 100.2 mmHg (3200 m), 2 days PiO_2_ = 97.9 mmHg (3400 m), 4 days ♦ Normoxia	♦ Participants received the same food menu	♦ Appetite ♦ Ghrelin ♦ PYY ♦ GLP-1 ♦ Average daily energy intake	♦ **Appetite** **⇔** ♦ **Ghrelin** ⇔ ♦ **PYY** ⇔ ♦ **GLP-1** **Before meal** - The post-test in the normoxia trial increased by 18% compared to the pre-test ♦ **Average daily energy intake** - Hypoxia ↓ 13% than normoxia
Morishima et al., 2016 [52]	♦ 8 healthy males ♦ Age: 21 ± 0.6 years ♦ BMI: 22.4 ± 2.4 kg/m^2^	♦ Randomized and crossover ♦ Acute	♦ Hypoxia: FiO_2_ = 15.0% ♦ Normoxia	♦ First meal (enter the laboratory for 1 h) - total calories: 3117 kJ (68.4% carbohydrate, 10.1% protein, 21.5% fat) ♦ Second meal (enter the laboratory for 4 h) - total calories: 3059 kJ (66.9% carbohydrate, 10.1% protein, 23.0% fat)	♦ Appetite ♦ Ghrelin ♦ GLP-1 ♦ Leptin	♦ **Appetite** ⇔ ♦ **Ghrelin** ⇔ ♦ **GLP-1** ⇔ ♦ **Leptin** ⇔
Debevec et al., 2014 [53]	♦ 11 healthy males ♦ Age: 27 ± 6 years ♦ BMI: 23.7 ± 3 kg/m^2^ ♦ VO_2max_: 44.3 ± 6.1 mL/kg/min	♦ Randomized and crossover ♦ Intervention duration: 21 days	♦ NBR ♦ HAMB - FiO_2_ = 14% - PiO_2_ = 90 mmHg - ambulatory confinement ♦ HBR - - FiO_2_ = 14% - - PiO_2_ = 90 mmHg	♦ Daily diet: total energy composition (fat ~30%, carbohydrate ~55%, protein ~15%, sodium intake < 3500 mg/day	♦ Appetite ♦ Energy intake	♦ **Appetite** ⇔ ♦ **Energy intake** - HAMB ↑ 8% than NBR - HAMB ↑ 9% than HBR
Matu et al., 2017 [33]	♦ 12 (9 males and 3 females) British Military volunteered participants ♦ Age: 28 ± 4 years ♦ BMI: 23.0 ± 2.1 kg/m^2^	♦ Completed 14-day trek in the Himalayas ♦ Altitude: ~1100 m increased to 5140 m	♦ Stay in Nepal for 3 days before the trek ♦ Rest day: camp 1; day 7 at 3619 m ♦ Rest day: camp 2; day 10 at 4600 m ♦ Rest day: camp 3; day 12 at 5140 m	♦ Daily diet: 49.0 ± 6.6 % carbohydrate, 36.3 ± 6.2% fat, 4.7 ± 2.6% protein	♦ Appetite ♦ Energy intake ♦ Ghrelin	♦ **Appetite** - 5140 m ↓ 22% than baseline - 5140 m ↓ 25% than 3619 m - 5140 m ↓ 18% than 4600 m ♦ **Energy intake** - 3619 m ↓ 26% than baseline - 5140 m ↓ 28% than baseline ♦ **Ghrelin** - 3619 m ↓ 13% than baseline - 4600 m ↓ 13.7% than baseline
Aeberli et al., 2013 [34]	♦ 25 healthy and experienced mountaineers (10 females and 15 males) ♦ Age: 43.8 ± 9.5 years ♦ BMI: 23.8 ± 2.2 kg/m^2^	♦ 4-day experiment ♦ Rapid ascent to 4559 m	♦ All examinations were performed at low (446 m) and high altitudes (2980 m and 4559 m)	♦ Meal: total calories were 400 Kcal (35% fat, 10% protein, 54% carbohydrate)	♦ Appetite ♦ Average dinner energy intake ♦ PYY	♦ **Appetite** **Before dinner** - 2980 m ↓ 31% than low altitude - 4559 m ↑ 62% than 2980 m **After dinner** - 4559 m ↑ 33% than 2980 m ♦ **Average dinner energy intake** - 2980 m ↓ 32% than low altitude - ⇔ between 4559 m and low altitude ♦ **PYY**⇔
Karl et al., 2018 [14]	♦ 17 unacclimatized males ♦ **Consumed higher protein (2.0 g/kg/d)** 9 males Age: 24 ± 7 years BMI: 25.5 ± 3.1 kg/m^2^ ♦ **Consumed standard-protein (1.0 g/kg/d)** 8 males Age: 23 ± 3 years BMI: 27 ± 4 kg/m^2^	♦ Randomized ♦ 21 days at sea level ♦ 22 days at high altitude (4300 m)		♦ Higher protein - 47% carbohydrate, 33% protein, 20% fat ♦ Standard-protein - 46% cabohydrate, 18% protein, 36% fat	♦ Appetite ♦ Ghrelin ♦ Leptin	♦ **Appetite** - ⇔ between sea level and high altitude **Acute hypoxia exposure (1st day) vs. sea level** - Standard-protein ↓ 33% - Higher protein ↓ 41% ♦ **Ghrelin** ⇔ between sea level and high altitude ♦ **Leptin** First day hypoxia exposure ↑ 18% than seven days at sea level
Abu Eid et al., 2018 [54]	♦ 40 male C57BL/6J mice	♦ Match for body weight, body composition, and basal blood glucose ♦ 3-month intervention	♦ Free access to conventional chow diet (chow ad libitum) ♦ Free access to high fat diet (HFD ad libitum) ♦ Free access to high fat diet under hypoxia (HFD + hypoxia) ♦ Restricted access to high fat diet (HFD restricted)	♦ High fat diet (60% of calories as fat)	♦ Energy intake ♦ Leptin	♦ **Energy intake** **First month** - HFD + hypoxia ↓ 30% than HFD *ad libitum* **Third month** - HFD + hypoxia ↓ 20% than HFD *ad libitum* ♦ **Leptin** - HFD + hypoxia ↑ 500% than chow *ad libitum* - HFD ad libitum ↑ 900% than chow *ad libitum*
Zaccaria et al., 2004 [32]	♦ 12 healthy males ♦ Age: 31.3 ± 6.4 years ♦ BMI: 22.88 ± 2.43 kg/m^2^	♦ Acute and long-term (15 days) treatment ♦ Altitude: 5050 m	♦ Acute and long-term (15 days) high altitude exposure	N/A	♦ Leptin	♦ **Leptin** - Acute high altitude exposure ↓ 35.6% than sea level - Long-term high altitude exposure ↓ 43.6% than sea level

↑ increase; ↓ decrease; ⇔ no difference. NBR: normoxic bed rest; HAMB: hypoxic ambulatory confinement; HBR: hypoxic bed rest.

According to previous literature, the subjective appetite appears to decrease at altitudes above 2500 m (FiO_2_ = 15.7%) with acute exposure conditions [14,33,34]. In contrast, no differences in subjective appetite appear to be induced at altitudes below 4500 m (FiO_2_ = 12.3%) or during prolonged acclimation [14,34,51,52,53]. Matu and colleagues recruited 12 British military personnel (eight men, four women; age: 28 ± 4 years; BMI: 23.0 ± 2.1 kg/m^2^) to spend 14 days climbing from sea level to an altitude of 5140 m (FiO_2_ = 11.3%), supplementing a fixed daily diet during the climb (49.0 ± 6.6% carbohydrate, 36.3 ± 6.2% fat, and 14.7 ± 2.6% protein) and they investigated the changes in appetite. These soldiers showed approximately a 28% reduction in energy intake compared to sea level, and there was trend of a reduction in hunger (*p* = 0.07) [33]. In another study [34], ten women and fifteen men (age: 43.8 ± 9.5 years; BMI: 23.8 ± 2.2 kg/m^2^) climbed to 2980 m (FiO_2_ = 14.5%) in two days and then to 4599 m (FiO_2_ = 12.1%) in two days. Breakfast was a fixed daily intake with a total of 400 calories (35% fat, 10% protein, and 54% carbohydrate). A casual diet was prepared for lunch and dinner. At 2980 m altitude, their subjective pre-dinner appetite was found to be approximately 31% lower than that at sea level. The subjective appetite was significantly associated with Lake Louise acute mountain sickness scores (*p* = 0.043, r = −0.468), but not with gender, age, or BMI [34]. The average energy intake at dinner was reduced by approximately 32%, whereas the appetite and energy intake at 4599 m altitude returned to a comparable level as at sea level. Blood concentrations induced by the appetite-suppressing hormone PYY were not significantly different at 2980 m and 4599 m [34]. Note that at 4600 m (FiO_2_ = 12.1%) energy intake began to return to levels similar to those at sea level, but during the short climb from sea level to an altitude above 2500 m, appetite and energy intake substantially decreased due to the maladaptation effects to the hypoxic environment. When climbing to higher altitudes, the body needs to adapt to a higher altitude environment again, resulting in the re-emergence of anorexia. These results suggest that when climbing to higher altitudes, the human body needs to adapt to an environment with more stress due to the rapid altitude change, making the hypoxia-induced anorexia reappear.

Although severe hypoxia may affect appetite by suppressing or promoting the secretion of appetite-suppressing hormones, excessive extreme conditions may also cause nausea, vomiting, dizziness, and other acute alpine illnesses, and such symptoms are likely to further generate negative effects on reducing energy intake [33,44,55,56]. Therefore, it is recommended that exposure to moderate hypoxia (a simulated altitude < 3000 m, FiO_2_ = 14.8%) is more practical for health promotion applications [52]. Although no significant changes in subjective appetite and appetite hormones were found, the study results by Morishima and Goto raise the consideration that exercise in a moderate hypoxic environment could have similar benefits to severe hypoxia [52]. Not only does exercise possibly reduce the risk of acute mountain sickness, it also maintains the post-exercise dietary intake, which could be of great benefit in promoting health and enhancing post-exercise recovery.

## 5. Acute Hypoxia Effects, Combined with Interval Exercise Challenges on Appetite, Related Regulatory Hormones, and Energy Intake

Exercise may cause exercise-induced anorexia nervosa [26,27], and an exposure to a hypoxic environment may produce high-altitude-induced anorexia nervosa [14,15,16,57]. Therefore, exercise training in a high-altitude environment is likely to have a synergistic, or even additive, effect on the severity of anorexia nervosa, but this phenomenon is still poorly understood. Currently, most studies on hypoxia combined with exercise have focused on the physiological mechanisms and benefits of high-altitude exercise training on athletic performance. Few studies have combined high-altitude environments with exercise challenges to observe appetite and the related regulatory hormonal changes. Furthermore, from the literature review above, it is clear that high-intensity exercise exhibits even greater impacts on appetite regulatory hormone secretion and energy intake compared to continuous exercise [27,30]. We have list the relevant literature on the effects of hypoxic combined interval exercise challenge on appetite regulation and energy intake in Table 3.

HIIT, which consists of high-intensity and low-intensity (or rest) exercise elements, is an effective health promotion and time-saving exercise strategy [8], which is widely used by athletes to enhance the efficiency of energy systems and exercise performance [9,10].

HIIT is not only widely used by athletes to enhance sports performance, but it has also become increasingly popular among the general population in recent years due to its time-saving features [58]. From 2016 to 2020, HIIT was ranked in the top 10 of the American College of Sports Medicine (ACSM) Global Fitness Trends Survey, which shows the high acceptance of this exercise strategy among the general population [59]. However, to date, only a few studies have examined the effects of combining HIIT and hypoxia on appetite and the related hormonal changes [60,61], and most studies regarding the hypoxic environment have focused on continuous exercise [16,55,56,62,63].

**Table 3:** Summary of the related studies on acute hypoxia effects combined with interval exercise on appetite and energy intake.

Authors (Years)	Subject (Human)	Experimental Design	Experimental Intervention	Dietary Control Methods	Measurement	Major Findings (Δ% Change)
Matu et al., 2017 [56]	♦ 12 healthy males ♦ Age: 30 ± 9 years ♦ BMI: 24.4 ± 2.7 kg/m^2^	♦ Randomized and crossover ♦ Acute	♦ 60 min (50% VO_2 max_) treadmill walk at sea level, 2150 m, and 4300 m	♦ Standardised evening meal: 1037 kcal, 57% carbohydrate, 28% fat, 15% protein ♦ Standardised breakfast: 322 kcal, 72% carbohydrate, 17% fat, 11% protein ♦ An ad libitum buffet meal was consumed 1.5 h after exercise	♦ Appetite ♦ Ghrelin ♦ GLP-1 ♦ Energy intake	♦ **Appetite** During exercise - 4300 m ↓ 25% than 2150 m After exercise - 4300 m ↓ 27% than 2150 m - 4300 m ↓ 33% than sea level ♦ **Ghrelin AUC** During exercise - 4300 m ↓ 30% than sea level After exercise - 4300 m ↓ 58% than sea level - 4300 m ↓ 56% than 2150 m ♦ **GLP-1** ⇔ ♦ **Energy intake** - 4300 m ↓ 49% than 2150 m
Bailey et al., 2015 [60]	♦ 12 physically active males (>150 min/wk of moderate-to-vigorous physical activity) ♦ Age: 21.6 ± 2 years ♦ BMI: 23.5 ± 2.0 kg/m^2^	♦ Four random order ♦ Acute	♦ MIE-normoxia ♦ MIE-hypoxia ♦ HIIT-normoxia ♦ HIIT-hypoxia ♦ MIE (50 min, 70% VO_2max_ running) ♦ HIIT (50 min, 7 min 70% VO_2max_ warm-up and cool down; 6 min * 3, 90% VO_2max_ running; 6 min* 3, 50% VO_2max_ active recovery) ♦ Hypoxic environment (2980 m)	♦ Standardised breakfast: 494 kcal, 78% carbohydrate, 16% protein, 6% fat ♦ Lunch meal (45 min post-exercise): 741 kcal, 74.5% carbohydrate, 21% protein, 4.5% fat	♦ Appetite ♦ PYY ♦ Ghrelin ♦ GLP-1	♦ **Appetite** - Hypoxia condition ↓ 22% than normoxia condition - No difference between in HIIE and MIE ♦ **PYY AUC** - HIIE-hypoxia ↑ 6% than MIE-hypoxia - HIIT-hypoxia ↑ 7% than HIIT-normoxia ♦ **Ghrelin AUC** After exercise - Hypoxia condition ↓ 13% than normoxia condition **During whole experiment** - Hypoxia condition ↓ 10% than normoxia condition ♦ **GLP-1** ⇔
Kojima et al., 2019 [61]	♦ 12 female athletes ♦ Age: 20.8 ± 0.2 years ♦ BMI: 21.8 ± 0.4 kg/m^2^	♦ Randomized and crossover ♦ Acute	♦ HIIT-normoxia (FiO_2_ = 20.9%, 2 sets, 6 s * 8 maximal sprint, 30 s rest, 10 min rest between sets) ♦ HIIT-hypoxia (FiO_2_ = 14.5%, 3000 m, 2 sets, 6 s * 8 maximal sprint, 30 s rest, 10 min rest between sets) ♦ Rest in normoxia	N/A	♦ Appetite ♦ Ghrelin ♦ GLP-1 ♦ Energy intake	♦ **Appetite** ⇔ ♦ **Ghrelin** **HIIT-normoxia** - Immediately post-exercise ↓ 39% than pre-exercise - 30 min post-exercise ↓ 52% than pre-exercise **HIIT-hypoxia** - Immediately post-exercise ↓ 40% than pre-exercise - 30 min post-exercise ↓ 48% than pre-exercise ♦ **GLP-1**⇔ ♦ **Energy intake** - HIIT-hypoxia ↓ 16% than rest - HIIT-normoxia ↓ 21% than rest
Debevec et al., 2016 [55]	♦ 11 healthy males ♦ Age: 27 ± 6 years ♦ BMI: 23.7 ± 3 kg/m^2^ ♦ VO_2max_: 44.3 ± 6.1 mL/kg/min	♦ Randomized and crossover ♦ Each designated condition: 21 days	♦ NBR ♦ HAMB - FiO_2_ = 14% - PiO_2_ = 90 mmHg - Moderate-intensity exercise (30 min * 2/day) ♦ HBR - FiO_2_ = 14% - PiO_2_ = 90 mmHg	♦ Dietary energy intake: 54% carbohydrate, 30% fat, 16% protein	♦ Appetite ♦ Ghrelin ♦ PYY ♦ GLP-1 ♦ Leptin ♦ Energy intake	♦ **Appetite** - Postprandial in NBR trial ↓ 23% than in pre-test ♦ **Ghrelin** - Post-test in HAMB ↑ 9% than in pre-test - Post-test in HBR ↑ 12% than in pre-test ♦ **GLP-1** ⇔ ♦ **Leptin** - Post-test in HAMB ↓ 23% than in pre-test ♦ **Energy intake** **Compared to target energy intake** - HAMB ↓ 14% - NBR ↓ 5% - HBR ↓ 6%
Wasse et al., 2012 [16]	♦ 10 healthy young males ♦ Age: 24 ± 3 years ♦ BMI: 24.8 ± 2.4 kg/m^2^	♦ Randomized and four-way crossover ♦ Acute	♦ Control-normoxia ♦ Control-hypoxia (FiO_2_: 12.7%, 4000 m) ♦ Exercise-normoxia (60 min, 70% VO_2max_ treadmill run) ♦ Exercise-hypoxia (FiO_2_ : 12.7%, 4000 m, 60 min 70% VO_2max_ treadmill run)	♦ Standardized meal: 65% carbohydrate, 27% fat, 8% protein, 42 kJ/kg body mass	♦ Appetite ♦ Ghrelin ♦ PYY ♦ Energy intake	♦ **Appetite** ⇔ ♦ **Ghrelin AUC** - Exercise-normoxia ↓ 15% than control-normoxia - Control-hypoxia ↓ 18% than control-normoxia - Exercise-hypoxia ↓ 10% than exercise-normoxia - Exercise-hypoxia ↓ 5% than control-hypoxia ♦ **PYY** - Exercise-normoxia ↑ 8% than control-normoxia - Exercise-hypoxia ↑ 10% than control-hypoxia ♦ **Energy intake** - Hypoxia condition ↓ 31% normoxia condition
Debevec et al., 2014 [62]	♦ 14 healthy males ♦ **Exercise group** 8 participants Age: 25.8 ± 2.4 years BMI: 22.9 ± 1.2 kg/m^2^VO_2peak_: 42.6 ± 6.1 mL/kg/min ♦ **Sedentary group** 6 participants Age: 24.8 ± 3.1 years BMI: 22.3 ± 2.5 kg/m^2^VO_2peak_: 42.2 ± 5.0 mL/kg/min	♦ Randomized ♦ 10-day intervention	♦ Exercise group (2*60 min bicycle training; exercise intensity: 50% heart rate, 140 ± 8 beats/min) ♦ Sedentary group ♦ FiO2 = 0.139 ± 0.003%, ~4000 m simulated altitude	N/A	♦ Ghrelin ♦ PYY ♦ GLP-1 ♦ Energy intake	♦ **Ghrelin** ⇔ ♦ **PYY** ⇔ ♦ **GLP-1** ⇔ ♦ **Energy intake** ⇔
Morishima et al., 2014 [63]	♦ 20 sedentary subjects ♦ **HYPO** 9 participants Age: 30 ± 2 years BMI: 25.6 ± 1.2 kg/m^2^ VO_2max_: 34.9 ± 2.2 mL/kg/min FiO_2_: 15% ♦ **NOR** 11 participants Age: 32 ± 3 years BMI: 25.4 ± 0.9 kg/m^2^VO_2max_: 34.5 ± 2.2 mL/kg/min FiO_2_: 20.9%	♦ Randomized ♦ 28-day intervention	♦ HYPO ♦ NOR ♦ Exercise: 60 min/day, 3 times/week, 55% VO_2max_ cycling	♦ 30 min post-exercise: jelly-type test meal (600 kcal; 65% carbohydrate, 15% protein, 20% fat)	♦ Appetite ♦ Ghrelin ♦ Leptin ♦ GLP-1 ♦ 3-day diet record	♦ **Appetite** ⇔ ♦ **Ghrelin** ⇔ ♦ **Leptin** **Fasting condition** - Post-test in NOR group ↓ 25% than in pre-test **Postprandial AUC** - Post-test in HYPO group ↓ 16% than in pre-test - Post-test in NOR group ↓ 30% than in pre-test ♦ **GLP-1** **Fasting condition** - Post-test in NOR group ↑ 43% than in pre-test **Postprandial AUC** - Post-test in NOR group ↑ 300% than in pre-test ♦ **3-day diet record** ⇔

↑ increase; ↓ decrease; ⇔ no difference. MIE: continuous moderate-intensity exercise; HIIT: high-intensity interval training; NBR: normoxic bed rest; HAMB: hypoxic ambulatory confinement; HBR: hypoxic bed rest; NOR: normoxic training; HYPO: hypoxic training; NOR: normoxic training; HYPO: hypoxic training.

Acute exposure to high altitude (> 3,500 m) is associated with marked changes in appetite regulation, but the combined effects of the hypoxic environment and HIIT on physiological mechanisms underlying appetite regulation have not been completely studied. Here, we compile recent studies focusing on the acute effects of HIIT on appetite in a hypoxic environment [60,61], in an attempt to understand the effects of low ambient oxygen concentration and HIIT on appetite and its regulatory hormonal responses. Bailey et al. (2015) used a randomized cross-over design to test the effects of four different exercise states on appetite, including: (1) normoxic moderate-intensity exercise (normoxic MIE; intensity: 70% VO_2_ max; duration: 50 min); (2) hypoxic MIE (FiO_2_ = 14.5%); (3) normoxic high-intensity interval training (normoxic HIIT; intensity: 90% VO_2_ max for 3 min plus 50% VO_2_ max for 3 min, repeated for 6 sets of cycles); and (4) hypoxic HIIT (FiO_2_ = 14.5%). In this study [60], a fixed breakfast (494 ± 27 calories, with 78% carbohydrate, 16% protein, and 6% fat) was given 2 h before each trial, and a post-exercise meal (741 ± 40 calories, 74.5% carbohydrate, 21% protein and 4.5% fat) was given at the end of the workout. Additionally, venous blood was collected, and the subjective appetite was assessed 2 h before exercise, as well as at the start of exercise, immediately after exercise, 45 min, 60 min, and 105 min after exercise. The authors found that an acute exercise challenge with hypoxia resulted in a rapid decrease in appetite and the suppression of circulating acylated-ghrelin concentrations, and no difference between the conditions of PYY and GLP-1 was observed during exercise. However, the impacts of an acute exercise challenge on appetite appeared to be similar between the two exercise modes [60]. Another study on HIIT in a hypoxic environment was conducted with female collegiate hockey players, who were tested in three different trials, including (1) a normoxic interval sprint exercise (ISE: 8 × 6 s maximal sprints with 30 s intervals for two rounds); (2) hypoxic ISE (FiO_2_ = 14.5%); and (3) a sedentary controlled trial. Blood was collected and the subjective appetite was evaluated before, immediately after, and 30 min after exercise, and a buffet-style breakfast was given to observe participants’ energy intake [61]. The authors reported that HIIT in both normoxic and hypoxic conditions resulted in comparable degrees of suppression of the subjective appetite, total energy intake (normoxic ISE: −21% vs. hypoxic ISE: −16%), and plasma acylated-ghrelin concentrations (normoxic ISE: −39% vs. hypoxic ISE: −40%), but GLP-1 was not significantly altered by ISE under different environmental oxygen concentrations. Although there was no significant difference in the total caloric intake between hypoxic and normoxic ISE, the reduction in caloric intake in both conditions was primarily due to a significant reduction in the amount of protein and fat intake [61].

However, it is still unclear as to whether a varied level of environmental hypoxia would elicit different degrees of an appetite response during HIIT. Although there is a lack of studies focused on the above issue, the existing studies used continuous exercise under varied hypoxic conditions, which may provide some implications on this aspect [16,55,56,62,63]. For example, Matu and colleagues compared the effects of 60 min of moderate continuous exercise (50% VO_2_ max) at sea level, 2150 m (FiO_2_ = 15.8%), and 4300 m (FiO_2_ = 11.7%) on appetite or appetite-regulating hormonal responses in a continuous exercise regime [56]. The acute effect of moderate continuous exercise (50% VO_2_ max) on the appetite regulation response was investigated at sea level, at simulated 2150 m (FiO_2_ = 15.8%) and at simulated 4300 m (FiO_2_ = 11.7%), and blood appetite regulatory hormones, subjective appetite scores, and post-exercise energy intakes were evaluated. The authors found that the decrease in subjective appetite and the decrease in the circulating ghrelin concentration was more pronounced when the ambient oxygen concentration decreased (i.e., sea level vs. 2150 m vs. 4300 m), but the decrease in total energy intake was equivalent across environmental conditions. Furthermore, there were no significant changes in circulating GLP-1 levels during moderate continuous exercise under hypoxic or normoxic conditions [56].

Although both of the above studies reported similar suppression of subjective appetite and famine by hypoxia or HIIT [60,61], the combination of hypoxia and HIIT did not seem to have additive effects. It is important to emphasize that there are still many experimental design diversities between these two hypoxic HIIT studies, which may affect the practical application of these results. These include gender differences in participants, duration of hypoxia exposure (5 h vs. 1 h), and type of intermittent exercise (longer intervals vs. shorter maximal sprints). Therefore, in practice, because of the paucity of available literature and the wide variation in experimental design, there is no conclusive evidence as to whether hypoxia and HIIT exercise have additive effects on appetite suppression.

## 6. Concluding Remarks and Suggestions for Future Research

In a normoxic environment, an acute exercise challenge seems to have no effect on subjective appetite, but there is still a significant impact on appetite-regulatory hormones. The possible reason for the above situation is that participants with professional exercise training experience (e.g., recreational sports participants or athletes) are already accustomed to the training intensity and, therefore, do not feel any loss of appetite, but it does not mean that their objective energy intake is not negatively affected. In addition, the higher the intensity of exercise and the longer the duration, the more likely it is to cause an exercise-induced loss of appetite and changes in appetite-regulatory hormones. On the other hand, studies have shown that gender, body fat, and hydration would make a difference in subjective appetite, energy intake, and the related regulation of appetite hormones.

An exposure to a hypoxic environment can lead to a decreased appetite, resulting in a decrease in subjective appetite and a negative effect on appetite-regulatory hormone regulation. However, most of the current research on hypoxia with exercise has investigated the benefits of hypoxic exercise training on exercise performance and has described the physiological mechanisms associated with the performance-enhancing benefits of exercise. On the contrary, there are not sufficient studies to provide details for the possible negative effects of combining hypoxia and HIIE on appetite. Furthermore, the studies involving the hypoxic factor have mostly examined continuous exercise as the mode of exercise. However, to date, only a few studies have examined the effects of combining the popular HIIT with a hypoxic environment on appetite changes. Although appetite-regulatory hormones, subjective appetite, and energy intake may be affected by exercise, hypoxia, or hypoxic exercise, we believe that the energy intake should be the main observable indicator in future studies on environmental and exercise interventions, because energy intake may directly affect the recovery effect after exercise. In addition, the possible effects of appetite regulation hormones and subjective appetite, as a regulatory mechanism, should not be ignored.

Since the specific hypoxic training modes, combined with high-intensity training, were effective in improving sports performance, this training mode, which integrates a hypoxic factor into the high-intensity training, appears to interfere with the desire of athletes’ diets or the desire to take nutritional supplements. This, in turn, affects and perturbs the recovery efficiency after exercise. Therefore, more research evidence may be needed to clarify how to maintain the appetite of trainees and conduct efficient sports nutrition supplementation strategies after hypoxic exercise training.

## Data Availability

Not applicable.
